# Function of the Human Cingulate Cortex: A Brainnetome Atlas‐Based Study via Cortical Electrical Stimulation in Patients With Epilepsy

**DOI:** 10.1002/cns.70768

**Published:** 2026-01-23

**Authors:** Qinqin Deng, Mengyang Wang, Guanpeng Chen, Xiongfei Wang, Zhaofen Yan, Huajun Yang, Yujiao Yang, Minghui Wang, Mengyi Guo, Zhonghua Xiong, Nan Guan, Jian Zhou, Yuguang Guan, Guoming Luan, Tianfu Li, Jing Wang

**Affiliations:** ^1^ Department of Neurology Sanbo Brain Hospital, Capital Medical University Beijing China; ^2^ Department of Neurosurgery Sanbo Brain Hospital, Capital Medical University Beijing China; ^3^ Beijing Key Laboratory of Behavior and Mental Health, School of Psychological and Cognitive Sciences Peking University Beijing China; ^4^ IDG/McGovern Institute for Brain Research Peking University Beijing China; ^5^ Beijing Key Laboratory of Epilepsy Sanbo Brain Hospital, Capital Medical University Beijing China; ^6^ Beijing Institute for Brain Disorders Capital Medical University Beijing China; ^7^ Laboratory for Clinical Medicine Capital Medical University Beijing China

**Keywords:** Brainnetome atlas, cingulate cortex, cortical electrical stimulation, drug‐resistant epilepsy, functional mapping

## Abstract

**Objective:**

This study aimed to systematically map the functional organization of the cingulate gyrus using cortical electrical stimulation (CES) guided by the Brainnetome Atlas.

**Methods:**

We retrospectively analyzed CES data from 234 patients with drug‐resistant epilepsy who underwent stereoelectroencephalography (SEEG) implantation in the cingulate cortex. A total of 1141 stimulation sites across seven cingulate subregions (A23d, A24rv, A32p, A23v, A24cd, A23c, A32sg) were examined. Responses were categorized into somatosensory, motor, autonomic, vestibular, visual, emotional, memory, and pain‐related phenomena.

**Results:**

Key findings included: (1) Somatosensory responses (*n* = 99) were widely distributed, with motor responses localized to middle‐posterior subregions (A23c/A24rv/A24cd); (2) Autonomic (*n* = 58) and emotional (*n* = 15) responses showed broad distribution, with ventral MCC (A24rv) as an affective hub; (3) Pain responses (*n* = 30) involved both anterior (affective) and posterior (spatial) subregions; (4) Memory deficits (*n* = 8) specifically localized to posterior cingulate (A23d); (5) Visual (*n* = 13) and vestibular (*n* = 51) responses clustered in posterior and middle‐posterior regions, respectively.

**Conclusion:**

This study provides a systematic functional mapping of the cingulate gyrus using the Brainnetome Atlas, demonstrating its integrated role in diverse neurological functions. The findings advance understanding of cingulate pathophysiology and have implications for surgical planning in epilepsy.

## Graphical abstract

This study provides a systematic functional mapping of the human cingulate cortex using cortical electrical stimulation guided by the Brainnetome Atlas. Key findings include distinct functional specializations across seven cingulate subregions. The results demonstrate the cingulate gyrus's integrated role in diverse neurological processes, offering insights for epilepsy surgery planning.

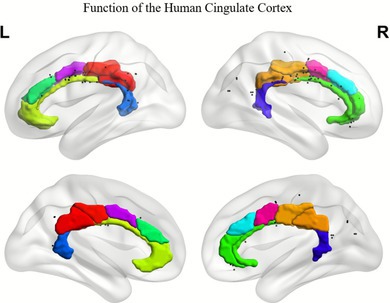

## 1 Introduction

The cingulate cortex (CC) is a crescent‐shaped region of the cerebral cortex situated on the medial surface of the cerebral hemispheres, bounded by the cingulate sulcus superiorly and the corpus callosum inferiorly [1]. The cingulate gyrus exhibits a complex subregional organization, which can be delineated using various methods including cytoarchitecture, myelination patterns, immunohistochemistry, functional magnetic resonance imaging (fMRI), and receptor distribution [2, 3, 4, 5]. The human Brainnetome Atlas, which comprises 210 cortical and 36 subcortical subdivisions, provides a fine‐grained, cross‐validated map incorporating both anatomical and functional connectivity data. Based on this atlas, the cingulate gyrus is subdivided into seven subregions (A23d, A24rv, A32p, A23v, A24cd, A23c, A32sg) [6].

Electrical cortical stimulation (ECS) is a powerful tool for investigating the human cingulate cortex. It is routinely used for functional mapping in patients with refractory epilepsy, as it helps both in localizing epileptogenic zones and elucidating cortical functions. However, ECS studies focusing on the cingulate cortex remain limited, particularly those involving large cohorts. Although recent studies [7, 8, 9] have employed ECS to explore cingulate functions to ECS in patients with epilepsy, these investigations utilized inconsistent parcellation schemes. To our knowledge, no study has yet systematically examined cingulate functions using the human Brainnetome Atlas. Therefore, this study aims to provide a detailed functional mapping of the cingulate gyrus based on this atlas.

In this study, we retrospectively analyzed ECS data from a relatively large cohort of patients with epilepsy who had been implanted with stereoelectroencephalography (SEEG) electrodes. By parcellating the cingulate gyrus according to the Brainnetome Atlas, we comprehensively mapped its functional organization to enhance the understanding of the roles of the cingulate cortex.

## 2 Patients and Methods

### 2.1 Patients

We retrospectively analyzed data from 234 patients with drug‐resistant epilepsy who underwent stereoelectroencephalography (SEEG) implantation involving the cingulate cortex at Sanbo Brain Hospital, Capital Medical University, between March 2015 and November 2021. The inclusion criteria were as follows: (1) availability of definitive anatomical and clinical data; and (2) implantation of at least one electrode within the cingulate cortex. Exclusion criteria included: (1) structural lesions (e.g., encephalomalacia or focal cortical dysplasia) at cingulate electrode sites; (2) pre‐existing psychiatric or cognitive impairments that could affect the documentation of ECS; or (3) identification of the epileptogenic zone within the cingulate cortex.

All patients underwent comprehensive presurgical evaluation including detailed medical history review, neurological examination, scalp video‐electroencephalography (VEEG), neuroimaging, and neuropsychological assessments. Electrode implantation strategies were determined through multidisciplinary discussion based on anatomo‐electroclinical hypotheses. Following intracranial video‐EEG monitoring, electrical cortical stimulation (ECS) was conducted after obtaining written informed consent. All procedures were reviewed and validated by a multidisciplinary team consisting of epileptologists, neurosurgeons, neuroelectrophysiologists, and neuropsychologists.

### 2.2 Electrode Implantation and vEEG Monitoring

Intracerebral multiple‐contact electrodes (8–16 contacts; length: 2 mm, diameter: 0.8 mm at 1.5 mm apart; Huake‐Hengsheng Medical Technology Co. Ltd.) were implanted with a robot‐assisted stereotactic surgery system. SEEG recordings were obtained with a Nicolet system (128 channels, sampling rate: 512 Hz; Thermo Nicolet Corporation). A postoperative computerized tomography (CT) scan was used to verify the absence of bleeding and the position of electrodes.

### 2.3 Localization of the Contacts

CT/MRI data fusion was performed, and the reconstructed images were digitally co‐registered with the “preoperative MRI dataset (using Brainstorm software). The fused datasets were visualized and reviewed in all three orientation planes to accurately determine the anatomical locations of the electrodes. Based on the three‐dimensional” integration of preoperative MRI and postoperative CT imaging, the cingulate cortex was parcellated according to the Brainnetome Atlas, and the corresponding electrode contacts were identified for subsequent analysis. The cingulate cortex was subdivided into seven subregions as defined by the Human Brainnetome Atlas: A23d, A24rv, A32p, A23v, A24cd, A23c, and A32sg. To account for interindividual anatomical variability, the relative positions of all electrode contacts were mapped onto standardized brain templates according to their symptom classifications.

### 2.4 Intracranial Electrical Stimulation Procedure

After the recording of spontaneous seizures, ECS was applied to elicit partial or complete electroclinical seizure patterns and to map functional cortical areas. Biphasic electrical stimuli were delivered using a Nicolet Cortical Stimulator (Middleton, WI) with the following parameters: pulse width of 0.3–0.5 ms, stimulus duration of 5 s, and frequency of 50 Hz. For each SEEG electrode, pairs of adjacent contacts (i.e., bipolar montage, also treated as a stimulated site in this study) were used for clinical inquiry. Stimulation was terminated upon either the onset of a clinical response or upon the detection of electrographic afterdischarges. The stimulation intensity ranged from 1 to 6 mA increased in 0.5 mA steps. The timing and site of stimulation were not disclosed to the patients. For the assessment of speech and memory functions, an active behavioral paradigm was employed during stimulation. In order to objectively measure speech production, fluency, and short‐term memory performance, patients were engaged in a counting task during stimulation. The stimulation outcomes were categorized into four categories: absence of response (stimulation up to 6 mA without eliciting clinical reactions), specific symptoms (including somatosensory, autonomic, vestibular, motor, pain, motor urges, speech, emotional, visual, memory, auditory, motor inhibition, hallucinatory, gustatory, and olfactory manifestations), afterdischarges, and epileptic seizures. This classification system allowed systematic functional mapping of the cingulate cortex through electrical stimulation.

### 2.5 Statistical Methods

Descriptive statistics were used to calculate response frequencies and distributions across subregions. Chi‐square (*χ* ^2^) tests were specifically applied to compare the occurrence of each functional response type between left and right hemispheric stimulation sites, with Fisher's exact test used when expected cell counts were less than 5 (significance threshold *p* < 0.05).

## 3 Results

**FIGURE 1:**
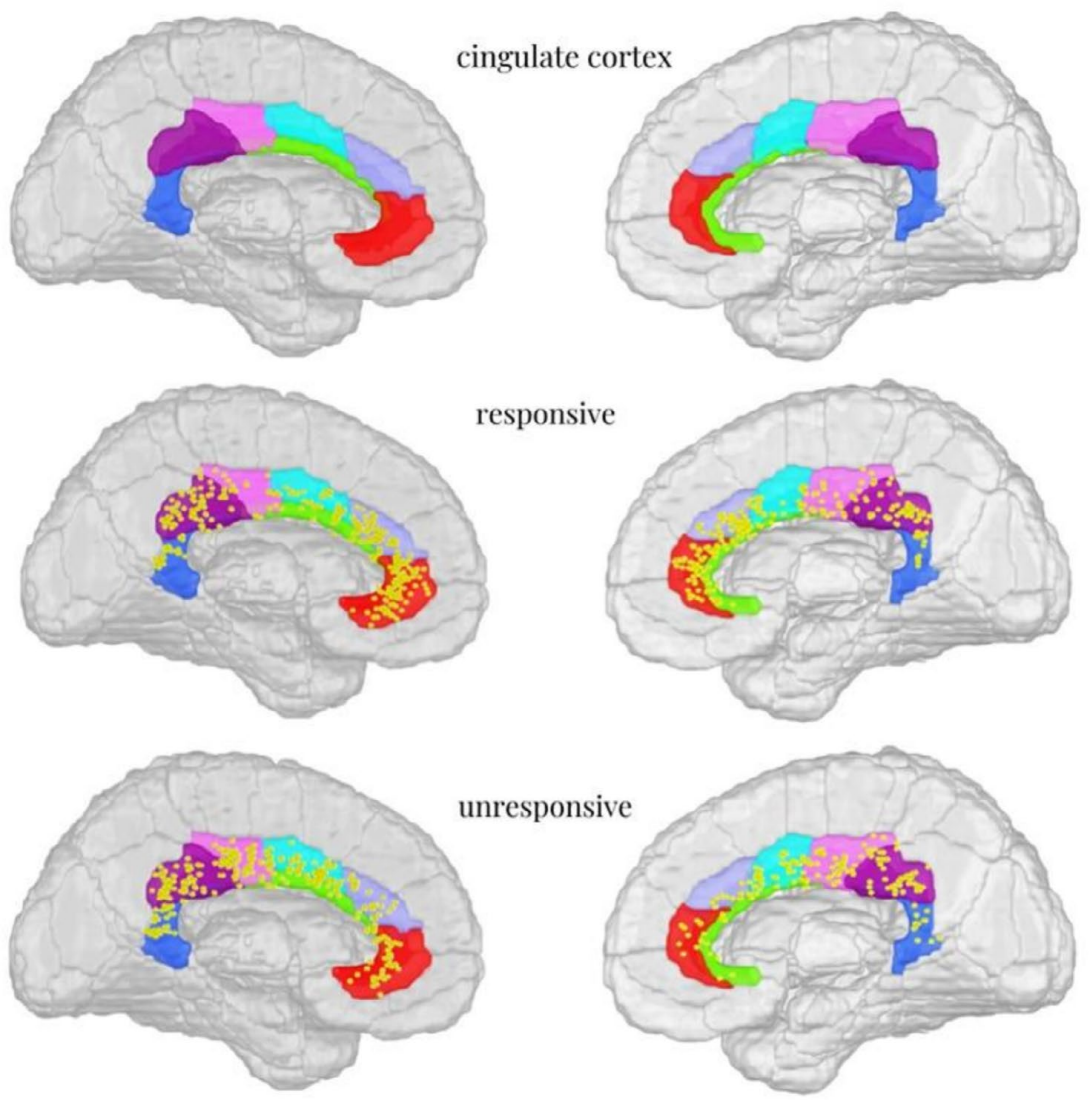
Distribution of responsive and unresponsive contacts across subdivisions of the cingulate cortex (Brainnetome Atlas). (1) cingulate cortex: According to the Brainnetome Atlas, the cingulate gyrus is subdivided into seven regions. Red color: A32sg, subgenual area 32; light purple color: A32p, pregenual area 32; green color: A24rv, rostroventral area 24; light blue color: A24cd, caudodorsal area 24; pink color: A23c, caudal area 24; purple color: A23d, dorsal area 23; blue color: A23v, ventral area 23. (2) responsive: The distribution of responsive contacts (3) unresponsive: The distribution of unresponsive contacts.

**FIGURE 2:**
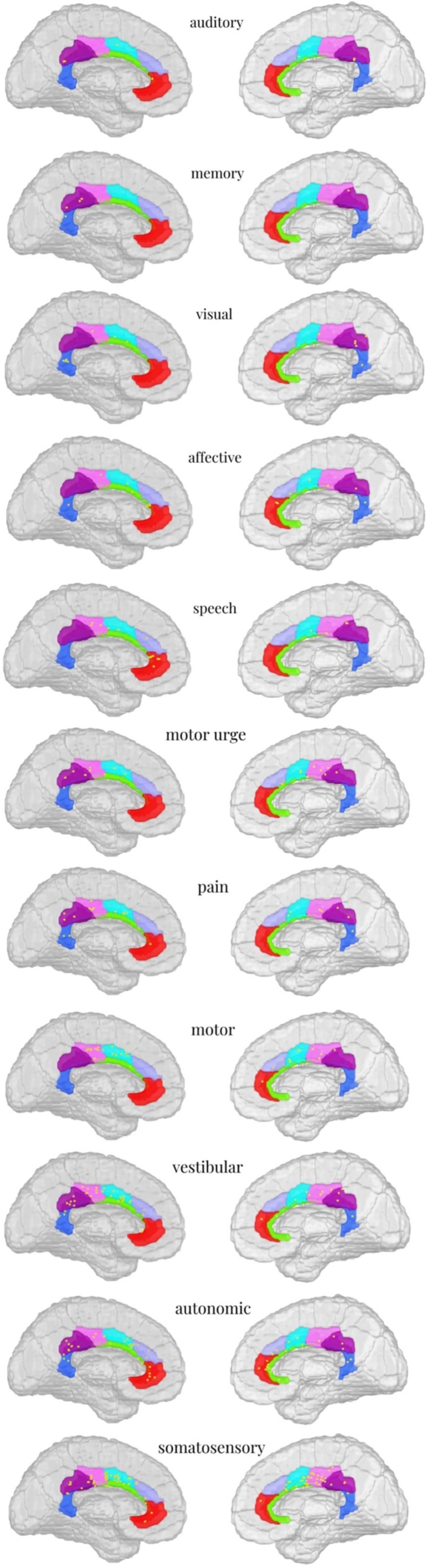
Distribution of different functions.

We analyzed a total of 234 patients (144 males and 90 females) with a mean age of 20.0 ± 10.2 years. Among these, 56 patients underwent bilateral electrode implantation, 103 received left‐sided implantation, and 75 received right‐sided implantation. The mean number of implanted electrodes per patient was 13.76, with stimulation conducted at 1 to 15 sites per patient (mean: 4.88 ± 3.18). Functional responses were assessed across 1141 stimulation sites (left hemisphere: *n* = 696; right hemisphere: *n* = 445; see Supporting Information for further details) (Figure 1). During stimulation, functional responses observed included somatosensory, autonomic, vestibular, motor, pain, motor urges, speech, emotional, visual, memory, auditory, motor inhibition, hallucinatory, gustatory, and olfactory manifestations (Table 1 and Figure 2). Absence of response was observed at 613 stimulation sites. Among the 528 responsive sites (stimulation intensity: 1–6 mA; mean threshold: 3.1 ± 1.8 mA), 67 sites elicited more than one type of response. The overall response rate was 46.28%.

**TABLE 1:** The statistics of ECS responses and results in cingulate cortex.

BNA	A23v			A23d			A23c			A24rv			A24cd			A32p			A32sg			Total			*p*
Lateral	L	R	T	L	R	T	L	R	T	L	R	T	L	R	T	L	R	T	L	R	T	L	R	T	
Contacts	77	42	119	141	85	226	100	67	167	55	118	173	92	33	125	106	51	157	125	49	174	696	445	1141	
Somatosensory	2	1	3	9	4	13	16	17	33	5	11	16	17	7	24	3	3	6	3	1	4	55	44	99	0.267
Autonomic	4	4	8	7	2	9	4	1	5	4	7	11	7	2	9	5	3	8	5	3	8	36	22	58	0.063
Vestibular	5	1	6	5	1	6	10	6	16	3	1	4	10	2	12	4	0	4	2	1	3	39	12	51	< 0.001*
Motor	0	0	0	1	0	1	9	3	12	1	3	4	8	2	10	2	0	2	0	1	1	21	9	30	0.024
Pain	3	1	4	4	2	6	6	3	9	1	0	1	6	1	7	1	1	2	1	0	1	22	8	30	0.011
Motor urge	1	0	1	3	2	5	3	3	6	1	3	4	2	2	4	0	0	0	0	0	0	10	10	20	1.000
Speech	0	0	0	0	0	0	4	2	6	1	1	2	2	1	3	3	0	3	3	0	3	13	4	17	0.034
Affective	1	1	2	0	1	1	1	0	1	4	1	5	1	1	2	1	1	2	0	2	2	8	7	15	0.774
Visual	4	2	6	0	3	3	1	1	2	1	0	1	1	0	1	0	0	0	0	0	0	7	6	13	0.764
Memory	2	0	2	5	1	6	0	0	0	0	0	0	0	0	0	0	0	0	0	0	0	7	1	8	0.065
Auditory	0	2	2	2	0	2	0	0	0	0	1	1	1	0	1	0	0	0	0	1	1	3	4	7	1.000
Motor inhibition	0	0	0	0	0	0	2	1	3	1	0	1	0	0	0	0	0	0	0	0	0	3	1	4	0.614
Hallucination	0	0	0	1	0	1	0	0	0	0	0	0	1	0	1	0	0	0	0	0	0	2	0	2	0.495
Taste	0	0	0	0	0	0	0	0	0	0	0	0	0	0	0	0	0	0	1	0	1	1	0	1	—
Olfaction	0	0	0	0	0	0	0	0	0	0	0	0	0	0	0	0	0	0	0	1	1	0	1	1	—
Others	4	2	6	0	0	0	4	2	6	5	10	15	10	2	12	3	2	5	2	0	2	28	18	46	0.118
Aura	0	2	2	1	0	1	2	3	5	0	2	2	2	3	5	1	1	2	4	0	4	10	11	21	
Seizure	1	0	1	0	0	0	3	0	3	1	1	2	1	3	4	1	1	2	1	0	1	8	5	13	
AD	26	10	36	42	24	66	23	9	32	5	4	9	5	2	7	9	2	11	13	3	16	123	54	177	
Unresponsive	30	22	52	69	49	118	28	24	52	26	79	105	32	11	43	78	36	114	91	38	129	354	259	613	
Responsive	83	48	131	149	89	238	116	75	191	59	124	183	106	39	145	111	50	161	126	51	177	750	476	1226	
Rate of no response (%)	38.96	52.38	43.70	48.94	57.65	52.21	28.00	35.82	31.14	47.27	66.95	60.69	34.78	33.33	34.40	73.58	70.59	72.61	72.80	77.55	74.14	50.86	58.20	53.72	

* *p* < 0.05.

### 3.1 Somatosensory

The most frequently evoked stimulation response in this study was somatosensory, with a total of 99 responses elicited (1–6 mA, mean: 2.5 ± 1.3 mA). These were primarily distributed in the following regions: A23v (*n* = 3), A23d (*n* = 13), A23c (*n* = 33), A24rv (*n* = 16), A24cd (*n* = 24), A32p (*n* = 6), and A32sg (*n* = 4). The predominant manifestations included limb numbness, electric shock‐like sensations, coldness, heat, itching, pulling sensations, and general discomfort.

### 3.2 Autonomic

A total of 58 responses were elicited (1–6 mA, mean: 3.1 ± 1.4 mA), mainly distributed in A23v (*n* = 8), A23d (*n* = 9), A23c (*n* = 5), A24rv (*n* = 11), A24cd (*n* = 9), A32p (*n* = 8), and A32sg (*n* = 8). The primary symptoms included palpitations, tachycardia, nausea, chest tightness, vomiting, dyspnea, and sweating.

### 3.3 Vestibular

A total of 51 responses were evoked (1–6 mA, mean: 3.1 ± 1.4 mA), predominantly in A23v (*n* = 6), A23d (*n* = 6), A23c (*n* = 16), A24rv (*n* = 4), A24cd (*n* = 12), A32p (*n* = 4), and A32sg (*n* = 3). The main manifestations were dizziness, vertigo (spinning sensation), and a heavy‐headed feeling.

### 3.4 Motor

A total of 30 responses were induced (1–6 mA, mean: 3.3 ± 1.6 mA), primarily in A23v (*n* = 0), A23d (*n* = 1), A23c (*n* = 12), A24rv (*n* = 4), A24cd (*n* = 10), A32p (*n* = 2), and A32sg (*n* = 1). The observed motor phenomena included limb rigidity, twitching, tonic deviation, eyelid myoclonus, rhythmic movements, and facial asymmetry (e.g., mouth deviation).

### 3.5 Pain

A total of 30 responses were recorded (1–6 mA, mean: 2.7 ± 1.5 mA), mainly distributed in A23v (*n* = 4), A23d (*n* = 6), A23c (*n* = 9), A24rv (*n* = 1), A24cd (*n* = 7), A32p (*n* = 2), and A32sg (*n* = 1). Pain was reported in multiple anatomical regions, including the head, knee, orbit, calf, and throat. Qualitative descriptors included pricking, distending, electric shock‐like, constrictive, and lancinating pain. Some cases were accompanied by autonomic symptoms, such as nausea and palpitations.

### 3.6 Motor Urge

A total of 20 responses were elicited (1–6 mA, mean: 3.3 ± 1.6 mA), primarily distributed in A23v (*n* = 1), A23d (*n* = 5), A23c (*n* = 6), A24rv (*n* = 4), A24cd (*n* = 4), A32p (*n* = 0), and A32sg (*n* = 0). The main manifestations included: sensations of body tilt, floating, or swaying; twitching, jerking, or pulsating sensations in limbs or specific body parts; and motor impulses such as the urge to make a fist.

### 3.7 Speech

Seventeen responses were elicited (2–6 mA, mean: 4.1 ± 1.3 mA), mainly distributed in A23v (*n* = 0), A23d (*n* = 0), A23c (*n* = 6), A24rv (*n* = 2), A24cd (*n* = 3), A32p (*n* = 3), and A32sg (*n* = 3). The primary manifestations included: dysfluent speech, counting cessation, slurred speech, slowed speech rate, and complete speech arrest.

### 3.8 Affective

Fifteen responses were elicited (1.5–5.5 mA, mean: 3.5 ± 1.3 mA), primarily distributed in A23v (*n* = 2), A23d (*n* = 1), A23c (*n* = 1), A24rv (*n* = 5), A24cd (*n* = 2), A32p (*n* = 2), and A32sg (*n* = 2). The main manifestations included: laughter, tension, fear, and fright.

### 3.9 Visual

Thirteen responses were elicited (1–6 mA, mean: 4.1 ± 1.7 mA), mainly distributed in A23v (*n* = 6), A23d (*n* = 3), A23c (*n* = 2), A24rv (*n* = 1), A24cd (*n* = 1), A32p (*n* = 0), and A32sg (*n* = 0). The primary manifestations included: visual oscillation, diplopia, micropsia (objects appearing distant), photopsia (flashes), visual hallucinations, blurred vision, and difficulty focusing.

### 3.10 Memory

Eight responses were elicited (1.5–6 mA, mean: 3.4 ± 1.4 mA), primarily distributed in A23v (*n* = 2), A23d (*n* = 6), A23c (*n* = 0), A24rv (*n* = 0), A24cd (*n* = 0), A32p (*n* = 0), and A32sg (*n* = 0). The main manifestation was forgetting numbers during counting tasks.

### 3.11 Auditory

Seven responses were elicited (1.5–5.5 mA, mean: 3.2 ± 1.5 mA), mainly distributed in A23v (*n* = 2), A23d (*n* = 2), A23c (*n* = 0), A24rv (*n* = 1), A24cd (*n* = 1), A32p (*n* = 0), and A32sg (*n* = 1). The primary manifestations included: unilateral or bilateral tinnitus and unilateral perception of “buzzing” sounds.

### 3.12 Other

Negative motor responses (*n* = 4) were distributed in A23c (*n* = 3) and A24rv (*n* = 1). Hallucinations (*n* = 2) were distributed in A23d (*n* = 1) and A24cd (*n* = 1). Gustatory responses (*n* = 1) were located in A32sg (*n* = 1). Olfactory responses (*n* = 1) were located in A32sg (*n* = 1).

### 3.13 Auras

Twenty‐one responses were elicited, primarily distributed in A23v (*n* = 2), A23d (*n* = 1), A23c (*n* = 5), A24rv (*n* = 2), A24cd (*n* = 5), A32p (*n* = 2), and A32sg (*n* = 4).

### 3.14 Seizures

Thirteen responses were elicited, mainly distributed in A23v (*n* = 1), A23d (*n* = 0), A23c (*n* = 3), A24rv (*n* = 2), A24cd (*n* = 4), A32p (*n* = 2), and A32sg (*n* = 1).

### 3.15 Afterdischarges

A total of 177 responses were elicited, primarily distributed in A23v (*n* = 36), A23d (*n* = 66), A23c (*n* = 32), A24rv (*n* = 9), A24cd (*n* = 7), A32p (*n* = 11), and A32sg (*n* = 16).

### 3.16 Non‐Responsive Sites

There were 613 stimulation sites that failed to elicit any responses, mainly distributed in A23v (*n* = 52), A23d (*n* = 118), A23c (*n* = 52), A24rv (*n* = 105), A24cd (*n* = 43), A32p (*n* = 114), and A32sg (*n* = 129).

### 3.17 Hemispheric Lateralization of Cingulate Cortex Functions

Statistical analysis revealed significant left‐hemispheric lateralization for vestibular responses (*p* < 0.001), while no significant hemispheric differences were found for: somatosensory (*p* = 0.267), autonomic (*p* = 0.063), motor (*p* = 0.024), pain (*p* = 0.011), emotional (*p* = 0.774), visual (*p* = 0.764), memory‐related (*p* = 0.065), speech (*p* = 0.034), motor urge (*p* = 1.000), auditory (*p* = 1.000), motor inhibition (*p* = 0.614), hallucinatory (*p* = 0.495).

## 4 Discussion

This study employed ECS of the cingulate cortex to investigate the functional specialization of distinct subregions within the cingulate cortex. The results indicate that the cingulate cortex is implicated in a wide range of processes, including somatic sensation, autonomic regulation, vestibular function, motor control, pain perception, emotional processing, vision, and memory.

### 4.1 Somatosensory and Motor

In this study, somatosensory responses were the most commonly elicited, with a total of 99 occurrences. These primarily included limb numbness, electric shock‐like sensations, thermal (cold/hot) sensations, itching, pulling sensations, and general discomfort. Somatosensory responses were widely distributed across the entire cingulate gyrus, which is consistent with previous studies [7, 9], indicating the cingulate cortex plays a fundamental role in the integration and processing of somatosensory information. Furthermore, high‐frequency stimulation of the anterior, middle, and posterior cingulate regions has been shown to induce alterations in body perception [10]. Motor responses in our study were predominantly elicited in the middle‐posterior cingulate regions (A23c, A24rv, A24cd), including manifestations such as limb rigidity, convulsions, deviation, eyelid twitching, swaying, and mouth corner deviation. Consistent with Caruana's findings, the highest density of activation sites was observed in the ventral and dorsal aMCC, where stimulation elicited various goal‐directed behaviors involving upper limbs or the entire body [7]. Additionally, it has been demonstrated that nearly all motor responses are evoked in the MCC [9].

### 4.2 Vestibular and Visual

Vestibular responses observed in this study primarily comprised dizziness, spinning sensations, and heavy‐headedness. These responses were distributed widely throughout the cingulate gyrus, with a higher prevalence observed in the middle to posterior regions, consistent with the findings reported by Caruana et al. [7] Visual responses included visual oscillation, diplopia, telescopic vision, flashes, hallucinations, blurring, and focusing difficulties. These were predominantly localized to middle‐posterior cingulate regions (A23v, A23d, A23c), with some responses in A24rv and A24cd. Caruana et al. [7] showed the caudal MCC contains the highest density of vestibular responses, while the PCC is principally associated with visual effects. Similarly, Xue et al. [9] observed vestibular responses mainly around the MCC‐PCC junction, whereas visual responses were predominantly induced within the PCC. Previous studies [11, 12] have further demonstrated PCC activation following vestibular stimulation, corroborating its involvement in vestibular processing. Smith et al. [13, 14] identified the cingulate sulcus visual area (CSv), located near the PCC‐MCC junction, as a region contributing to vestibular‐visual integration. Pelliccia et al. [15] reported ACC stimulation could elicit atypical responses including speech disturbances, vestibular symptoms, and nonspecific subjective experiences like confusion or “foggy” sensations. Our observation of sparse vestibular responses in the ACC warrants investigation into whether ACC directly processes vestibular input or mediates it indirectly via autonomic pathways.

### 4.3 Autonomic and Affective

Autonomic responses included palpitations, tachycardia, nausea, chest tightness, dyspnea, and sweating, and were observed across all cingulate subregions. Caruana et al. [7] reported predominant autonomic responses in pACC with occasional aMCC involvement, while Oane et al. [8] demonstrated a more uniform distribution of autonomic responses throughout the cingulate cortex, which aligns closely with our findings. Pelliccia et al. [15] and Xue et al. [9] both confirmed autonomic responses in MCC/PCC, indicating distributed autonomic representation. Emotional responses (15 instances) primarily involved laughter, tension, fear, and panic. These findings mirror those of Xue et al. [9] who reported that affective responses (predominantly fear and smiling) were clustered in the ACC and ventral MCC, with rare occurrences in the PCC. Similarly, our data indicated that emotional responses occurred throughout various cingulate regions, with the ventral MCC (A24rv) identified as a particularly prominent hotspot.

### 4.4 Memory

Memory‐related responses (8 instances) localized to posterior cingulate (A23v, A23d), manifesting as numeral forgetting during counting tasks, suggesting cingulate involvement in memory processes. Substantial evidence implicates the PCC in memory function: Minoshima et al. [16] found markedly reduced metabolic activity in the PCC metabolism in early Alzheimer's disease; Zhou et al. [17] demonstrated decreased functional and structural connectivity between the PCC and hippocampus in patients with mild cognitive impairment (MCI) and Alzheimer's disease (AD) using fMRI and DTI; Leech et al. [18] suggested that the PCC modulates global brain metastability, thereby influencing attentional focus and memory; furthermore, Natu et al. [19] showed that electrical stimulation of the PCC impaired episodic memory encoding, supporting the potential of deep cortical stimulation for memory modulation. These findings warrant further exploration of cingulate memory mechanisms.

### 4.5 Pain Processing

Pelliccia et al. [15] suggested that the organization of the pain matrix within the cingulate gyrus warrants further investigation. In the present study, we elicited a total of 30 pain responses distributed across the anterior, middle, and posterior cingulate regions. These responses manifested as pain in various locations (e.g., head, knees, orbits, calves, throat), characterized by qualities such as needle‐like, distending, electric‐shock‐like, constrictive, and knife‐cut sensations, and were frequently accompanied by autonomic symptoms such as nausea and palpitations. Substantial evidence supports the involvement of the anterior cingulate cortex's (ACC) in pain processing: Hutchison et al. [20] identified individual ACC neurons selectively responding to painful thermal and mechanical stimuli; functional imaging studies [21] show that painful heat stimuli induce significant activation in the contralateral ACC, secondary somatosensory cortex, and primary somatosensory cortex; Ingvar et al. [22] demonstrated ACC and ventral insula involvement in pain modulation; Xu et al. [23] found that ACC sensitization plays an important role in chronic pain; Moon et al. [24] showed the ACC is crucial for acute pain perception and neuropathic pain development, characterized by long‐term potentiation in pain pathways; Bliss et al. [25] reported altered synaptic plasticity in the ACC during chronic pain in animal models; Vogt et al. [26] noted that most studies on nociceptive processing in the cingulate cortex emphasize the ACC and MCC, as these regions mediate the affective component of pain. In contrast, research on the contribution of the posterior cingulate cortex (PCC) to pain processing remains limited. Vogt et al. [3] proposed that the PCC participates in visuospatial orientation, with its dorsal portion (dPCC) potentially directing attention toward both innocuous and noxious somatosensory stimuli and possibly sharing functions with the pMCC. Bentley et al. [27] used electrocorticography to demonstrate that both pMCC and dPCC exhibit short‐latency responses to nociceptive stimuli. Additionally, Niddam et al. [28] found that painful and non‐painful electrical muscle stimulation activates the caudal cingulate motor area and dPCC, suggesting that these regions are involved in orienting the body to sensory stimuli, including nociceptive inputs. Thus, the PCC may also contribute to pain localization. Together with our findings, these observations suggest that the cingulate gyrus participates in pain processing through a more extensive functional network than previously recognized.

### 4.6 Limitations

Several methodological and cohort‐related factors should be considered when interpreting our findings. First, over half of the stimulated sites (613/1141) elicited no observable responses. This may reflect the passive nature of our bedside stimulation paradigm, which is less sensitive to higher‐order cognitive functions such as conflict monitoring or error detection [9, 15], as well as limitations in stimulation parameters. While we used active tasks for speech and memory assessment, a more systematic task‐concurrent approach or multimodal imaging may better capture the full functional repertoire of the cingulate cortex. Second, our cohort included patients with diverse pathologies and a broad age range. Although the consistency of our topographic maps with prior studies supports the conserved functional architecture of the cingulate gyrus [7, 9], future studies in more homogeneous adult cohorts are needed to disentangle potential influences of pathology‐specific network reorganization and neurodevelopmental plasticity on functional mapping.

## 5 Conclusion

This study delineates multifaceted functional roles of the cingulate cortex in somatosensation, autonomic regulation, vestibular/visual processing, motor function, pain perception, emotion, and memory through direct electrical stimulation. By applying the Brainnetome Atlas for precise functional parcellation, we establish detailed anatomical‐functional correlations that refine the understanding of cingulate organization. Future studies should continue to elucidate the functional attributes of the cingulate cortex to enhance its clinical and physiological significance.

## Author Contributions

Q.D., J.W., T.L., and M.W. contributed to the conception and design of the study. All authors contributed to the acquisition and analysis of data. Q.D. contributed to drafting the manuscript and preparing the figures. G.C. contributed to interpreting the results. All authors reviewed and revised the manuscript for intellectual content.

## Funding

This work was supported by CAAE Epilepsy Research Fund‐UCB Fund (CU‐2023‐052), CAAE‐Neuracle Neuroelectrics Research Fund (CB‐2022‐017), STI2030‐Major Projects (2022ZD0205000), the National Natural Science Foundation of China (32020103009) and Capital's Health Development Research Fund (2024–2‐6011).

## Ethics Statement

Approval for this study was obtained from the Ethics Committee of Sanbo Brain Hospital, Capital Medical University.

## Conflicts of Interest

The authors declare no conflicts of interest.

## Supporting information


**Data S1:** cns70768‐sup‐0001‐Supinfo.docx.

## Data Availability

The data that support the findings of this study are available from the corresponding author upon reasonable request.
