# Supplementary material for: Function of the Human Cingulate Cortex: A Brainnetome Atlas‐Based Study via Cortical Electrical Stimulation in Patients With Epilepsy
Source: CNS Neurosci Ther. 2026 Jan 23;32(1):e70768. doi: 10.1002/cns.70768 (PMC12828339; doi:10.1002/cns.70768)
Supplement: Supplementary file 1 — Data S1: cns70768‐sup‐0001‐Supinfo.docx. [file CNS-32-e70768-s001.docx]

Supplementary Materials.List of patients investigated.

| Patient | Sex | Age | MRI abnormal lobes | Surgical approach | Pathology | Number of Electrodes | Side of Electrodes | Number of Contacts |
| --- | --- | --- | --- | --- | --- | --- | --- | --- |
| 1 | M | 5 | L Hippocampus | TR | FCD Ⅰb+HS | 7 | L | 7 |
| 2 | M | 24 | L Temporal-parietal-occipital | TR | Post-traumatic | 13 | L | 3 |
| 3 | F | 3 | R Temporal | / | Not available | 11 | R | 2 |
| 4 | F | 7 | L Frontal | TR | FCD Ⅰb | 11 | L | 3 |
| 5 | M | 15 | Negative | TR+BC | FCDⅠc+GMH | 15 | R | 2 |
| 6 | F | 28 | Negative | TR | FCD ⅡA | 12 | B | 1 |
| 7 | F | 31 | Negative | TR+BC | FCD Ⅱb | 14 | L | 1 |
| 8 | M | 17 | Negative | TR | Post-traumatic | 13 | B | 5 |
| 9 | M | 14 | Negative | TR+BC | FCDⅠb+GMH | 11 | R | 2 |
| 10 | M | 51 | Negative | VNS | Not available | 15 | L | 3 |
| 11 | F | 25 | L Hippocampus | TR | FCD Ⅰb | 11 | R | 1 |
| 12 | F | 30 | L Insula | TR | Post-traumatic | 9 | L | 3 |
| 13 | F | 18 | R Frontal | TR | FCD Ⅰb | 9 | R | 3 |
| 14 | M | 32 | Negative | RF-TC | Not available | 12 | L | 6 |
| 15 | F | 27 | L Frontal | / | Not available | 11 | L | 5 |
| 16 | F | 10 | Hypothalamic hamartoma | RF-TC | Not available | 8 | L | 8 |
| 17 | F | 27 | Negative | TR+BC | FCDⅡa+FCDⅠb | 13 | B | 5 |
| 18 | F | 25 | B Parietal | RF-TC | Not available | 13 | B | 3 |
| 19 | M | 31 | R Temporal | RF-TC | Not available | 9 | R | 3 |
| 20 | F | 26 | L Hippocampus | TR | FCDⅠb+HS | 12 | L | 5 |
| 21 | M | 17 | L Parietal | RF-TC | Not available | 8 | L | 1 |
| 22 | F | 19 | B Parietal-occipital | TR | Post-traumatic | 11 | B | 2 |
| 23 | M | 26 | B hippocampus | TR+BC | FCDⅠb+HS | 11 | B | 3 |
| 24 | F | 8 | Negative | TR | FCD Ⅱa | 10 | L | 5 |
| 25 | F | 6 | Negative | RF-TC | Not available | 12 | L | 7 |
| 26 | M | 30 | Negative | TR | FCD | 12 | L | 4 |
| 27 | M | 31 | L Frontal | RF-TC | Not available | 12 | B | 5 |
| 28 | M | 23 | Negative | RF-TC | Not available | 12 | B | 4 |
| 29 | M | 25 | Negative | TR | FCDⅠb | 11 | R | 1 |
| 30 | M | 15 | Negative | RF-TC | Not available | 9 | R | 7 |
| 31 | M | 27 | R Frontal-insula | / | Not available | 12 | L | 11 |
| 32 | M | 30 | R Temporal | / | Not available | 14 | R | 2 |
| 33 | F | 27 | R Frontal | TR | FCDⅡb | 6 | R | 6 |
| 34 | M | 10 | Negative | TR+BC | FCDⅠa | 12 | R | 5 |
| 35 | F | 19 | Negative | TR | FCD | 9 | R | 11 |
| 36 | F | 7 | Negative | VNS | Not available | 10 | L | 8 |
| 37 | M | 34 | Negative | RF-TC | Not available | 12 | L | 2 |
| 38 | F | 28 | L Temporal | TR | FCD Ⅰb+GMH | 12 | L | 2 |
| 39 | M | 19 | R Parietal-occipital | TD | Not available | 12 | R | 4 |
| 40 | M | 31 | L Temporal-parietal | TR+BC | FCDⅠb | 11 | L | 2 |
| 41 | F | 15 | L Frontal | / | Not available | 9 | L | 10 |
| 42 | M | 26 | R Frontal and B hippocampus | TR | FCDⅠc | 13 | L | 3 |
| 43 | M | 4 | Negative | TR | GMH | 10 | L | 8 |
| 44 | M | 23 | Negative | RF-TC | Not available | 10 | B | 1 |
| 45 | M | 28 | Negative | TR | FCDⅠb | 10 | R | 3 |
| 46 | F | 22 | R Frontal | TR+BC | Post-traumatic | 9 | R | 5 |
| 47 | M | 8 | L Temporal | RF-TC | Not available | 14 | L | 3 |
| 48 | F | 30 | R Parietal-occipital | VNS | Not available | 12 | B | 1 |
| 49 | M | 27 | Negative | TR | FCD Ⅰa | 8 | R | 1 |
| 50 | M | 6 | L Frontal | TR | FCD Ⅱa | 9 | L | 10 |
| 51 | F | 10 | R Frontal | TR | FCDⅠb | 11 | R | 9 |
| 52 | M | 6 | Negative | RF-TC | Not available | 12 | B | 4 |
| 53 | F | 5 | L Frontal | TR | FCDⅡb | 5 | L | 7 |
| 54 | F | 13 | R Temporal | TR | Ganglioglioma | 10 | R | 4 |
| 55 | M | 17 | R hippocampus | TR | FCD | 15 | R | 8 |
| 56 | M | 24 | Negative | VNS | Not available | 8 | L | 4 |
| 57 | M | 20 | Negative | TR | FCDⅡb | 10 | L | 9 |
| 58 | F | 26 | Negative | / | Not available | 10 | R | 11 |
| 59 | M | 5 | Negative | RF-TC | Not available | 12 | R | 7 |
| 60 | M | 26 | L Temporal | TR+BC | FCDⅠb | 12 | L | 11 |
| 61 | F | 2 | B Parietal-occipital | RF-TC | Not available | 15 | B | 15 |
| 62 | M | 15 | L Frontal | TR | FCDⅡa | 7 | B | 7 |
| 63 | M | 15 | Negative | VNS | Not available | 15 | R | 4 |
| 64 | M | 31 | L Temporal | TR | FCDⅠc | 14 | L | 4 |
| 65 | F | 35 | L Temporal-Parietal | TR+BC | FCD Ⅱa | 11 | B | 4 |
| 66 | F | 30 | L Frontal | TR | Encephalomalacia | 13 | B | 1 |
| 67 | F | 18 | R Temporal | TR | FCDⅠc | 12 | B | 2 |
| 68 | M | 18 | B hippocampus | TR | Unclassifiable | 11 | B | 1 |
| 69 | M | 12 | R Temporal-Parietal | RF-TC | Not available | 10 | R | 5 |
| 70 | F | 5 | Negative | RF-TC | Not available | 11 | L | 8 |
| 71 | F | 26 | Negative | RF-TC | Not available | 11 | B | 3 |
| 72 | F | 23 | L Temporal and R Frontal | TR | HS | 12 | L | 6 |
| 73 | F | 34 | R Parietal | TR | FCD | 10 | R | 3 |
| 74 | F | 23 | B Temporal-occipital | TR | FCD Ⅰb | 14 | B | 1 |
| 75 | M | 22 | L Hippocampus | TR | FCDⅠb+HS | 11 | B | 8 |
| 76 | M | 21 | R Temporal-parietal-occipital and L Parietal | / | Not available | 10 | R | 6 |
| 77 | F | 18 | R Frontal and R Parietal | TR | FCD Ⅱb | 15 | B | 4 |
| 78 | M | 26 | Negative | TR | FCDⅠb | 11 | B | 4 |
| 79 | M | 30 | Negative | TR | FCDⅠb | 13 | B | 1 |
| 80 | M | 19 | Negative | TR | FCDⅠb | 10 | B | 4 |
| 81 | M | 27 | Negative | TR | Unclassifiable | 14 | B | 6 |
| 82 | F | 29 | R Frontal | TR | FCDⅡb | 10 | R | 5 |
| 83 | F | 32 | B Parietal-occipital | RF-TC | Not available | 19 | B | 2 |
| 84 | F | 8 | Negative | TR+BC | FCDⅡb | 10 | R | 7 |
| 85 | M | 28 | white matter | TR | FCDⅠb | 12 | L | 9 |
| 86 | M | 10 | Negative | RF-TC | Not available | 13 | B | 4 |
| 87 | F | 23 | Negative | TR | FCDⅠb | 9 | R | 8 |
| 88 | M | 20 | B Parietal-occipital | RF-TC | Not available | 14 | B | 6 |
| 89 | M | 28 | Negative | TR | FCDIIa+GMH | 9 | L | 12 |
| 90 | F | 7 | Negative | TR | FCDⅠb | 11 | B | 6 |
| 91 | F | 16 | L Temporal | RF-TC | Not available | 9 | L | 6 |
| 92 | M | 22 | R Occipital | TR | Ganglioglioma | 12 | B | 2 |
| 93 | M | 30 | Negative | TR | FCDⅠb | 11 | L | 5 |
| 94 | M | 7 | R Parietal | RF-TC | Not available | 10 | R | 3 |
| 95 | M | 9 | Hypothalamic hamartoma | RF-TC | Not available | 4 | B | 3 |
| 96 | M | 31 | R hippocampus | TR | FCDⅠb+HS | 7 | R | 1 |
| 97 | F | 14 | Negative | RNS | Not available | 11 | L | 4 |
| 98 | F | 3 | Negative | TR | FCD Ⅱa | 10 | L | 2 |
| 99 | M | 16 | Negative | RF-TC | Not available | 10 | R | 3 |
| 100 | M | 8 | Negative | RF-TC | Not available | 11 | L | 4 |
| 101 | M | 9 | Negative | RF-TC | Not available | 14 | B | 3 |
| 102 | F | 39 | Negative | RF-TC | Not available | 15 | R | 2 |
| 103 | M | 28 | Negative | TR | FCDⅠb | 12 | L | 9 |
| 104 | M | 25 | Negative | RF-TC | Not available | 10 | B | 4 |
| 105 | M | 19 | R Parietal | RF-TC | Not available | 14 | R | 2 |
| 106 | M | 30 | L Temporal | TR | FCD）Ⅱa | 11 | L | 5 |
| 107 | M | 22 | Negative | RF-TC | Not available | 9 | R | 11 |
| 108 | M | 37 | Negative | TR | FCDⅠb | 14 | R | 4 |
| 109 | M | 5 | R Frontal | TR | FCDⅠb | 11 | R | 6 |
| 110 | M | 17 | Negative | TR | GMH | 11 | R | 2 |
| 111 | F | 9 | R Frontal-insula | TR | FCDⅡb | 7 | R | 3 |
| 112 | M | 3 | Negative | RF-TC | Not available | 9 | B | 3 |
| 113 | M | 19 | Negative | RF-TC | Not available | 13 | L | 8 |
| 114 | M | 18 | B Parietal-occipital | VNS | Not available | 16 | B | 2 |
| 115 | M | 32 | Negative | RF-TC | Not available | 11 | L | 9 |
| 116 | M | 13 | R hippocampus | RF-TC | Not available | 18 | B | 12 |
| 117 | F | 17 | Negative | TR | FCDⅠb | 8 | R | 2 |
| 118 | F | 12 | L Parietal-occipital | TR | Post-traumatic | 15 | B | 1 |
| 119 | M | 8 | Negative | VNS | Not available | 8 | R | 2 |
| 120 | M | 35 | R Parietal | RF-TC | Not available | 10 | R | 4 |
| 121 | F | 8 | R Frontal | TR | FCD Ⅱa | 7 | R | 1 |
| 122 | F | 30 | L Parietal | RF-TC | Not available | 11 | L | 4 |
| 123 | F | 9 | R Temporal | RF-TC | Not available | 12 | B | 12 |
| 124 | F | 18 | R Frontal | TR | Not available | 11 | L | 10 |
| 125 | M | 7 | R Parietal | RF-TC | Not available | 11 | R | 1 |
| 126 | M | 24 | Negative | TR | FCDⅡa | 16 | B | 5 |
| 127 | M | 34 | R Temporal | TR+BC | FCDⅠb+GMH | 13 | B | 2 |
| 128 | M | 31 | R Frontal-insula | TR | Not available | 11 | B | 10 |
| 129 | M | 22 | Negative | RF-TC | Not available | 13 | L | 3 |
| 130 | M | 23 | Negative | RF-TC | Not available | 7 | L | 5 |
| 131 | M | 29 | L Frontal | TR | FCD Ⅱb | 8 | L | 3 |
| 132 | M | 20 | R Temporal | TR | Encephalomalacia | 12 | R | 1 |
| 133 | F | 46 | Negative | TR | Unclassifiable | 13 | L | 1 |
| 134 | M | 29 | Negative | / | Not available | 11 | L | 2 |
| 135 | F | 7 | R Temporal | TR | FCD Ⅰb | 11 | R | 1 |
| 136 | M | 9 | L Frontal | TR | FCD Ⅱa | 11 | L | 8 |
| 137 | M | 30 | R Frontal-parietal | RF-TC | Not available | 10 | R | 6 |
| 138 | M | 25 | Negative | / | Not available | 9 | R | 3 |
| 139 | F | 27 | Negative | TR | FCDⅠb | 14 | L | 4 |
| 140 | F | 35 | R Temporal-Parietal | TR+BC | Unclassifiable | 12 | R | 5 |
| 141 | M | 19 | Ependyma | RF-TC | Not available | 12 | R | 5 |
| 142 | F | 13 | L Cingulate gyrus | TR | FCDⅡb | 10 | L | 7 |
| 143 | M | 12 | Negative | / | Not available | 8 | L | 10 |
| 144 | F | 38 | L Temporal | RF-TC | Not available | 18 | B | 4 |
| 145 | M | 13 | L Temporal | TR | Unclassifiable | 13 | L | 2 |
| 146 | M | 8 | L Hippocampus | TR | FCDⅠb +HS | 8 | L | 6 |
| 147 | M | 30 | Negative | TR | FCDⅡ+GMH | 8 | R | 2 |
| 148 | F | 13 | L Frontal | vns | Not available | 12 | L | 5 |
| 149 | M | 21 | Negative | RF-TC | Not available | 10 | L | 6 |
| 150 | M | 9 | R Frontal | RF-TC | Not available | 3 | R | 5 |
| 151 | M | 8 | L Frontal | TR | FCDⅡb | 8 | L | 7 |
| 152 | M | 42 | B hippocampus | RF-TC | Not available |  |  | 2 |
| 153 | F | 15 | R Temporal | TR+BC | Ganglioglioma | 11 | R | 3 |
| 154 | F | 30 | Subependymal | VNS | Not available | 14 | B | 2 |
| 155 | M | 9 | Negative | TR | FCDⅠb | 10 | L | 2 |
| 156 | F | 26 | Negative | RF-TC | Not available | 10 | L | 6 |
| 157 | M | 23 | Negative | TR | Encephalomalacia | 11 | R | 1 |
| 158 | M | 4 | R Frontal | VNS | Not available | 11 | L | 8 |
| 159 | F | 26 | Negative | TR | Unclassifiable | 10 | L | 4 |
| 160 | M | 25 | R Frontal | TR | FCD Ⅱa | 9 | B | 8 |
| 161 | F | 37 | Negative | TR | FCDⅠb | 12 | R | 2 |
| 162 | F | 29 | L Temporal-occipital | TR | Post-traumatic | 9 | L | 4 |
| 163 | M | 3 | Negative | RF-TC | Not available | 11 | R | 5 |
| 164 | F | 26 | Negative | TR | FCDⅠc | 13 | L | 4 |
| 165 | M | 8 | L Frontal | TR | FCD Ⅱa | 8 | L | 2 |
| 166 | M | 25 | Negative | RF-TC | Not available | 11 | L | 7 |
| 167 | M | 4 | Negative | TR | FCDⅠb型 | 12 | L | 3 |
| 168 | F | 21 | Negative | TR | FCD Ⅱa | 15 | B | 8 |
| 169 | M | 30 | Negative | TR | FCDⅠc | 13 | R | 1 |
| 170 | F | 30 | L Hippocampus | RF-TC | Not available | 13 | L | 2 |
| 171 | M | 14 | Negative | RF-TC | Not available | 14 | R | 9 |
| 172 | F | 13 | R hippocampus | TR+BC | FCDⅠb | 10 | R | 3 |
| 173 | M | 28 | Negative | TR | FCDⅠb | 10 | R | 10 |
| 174 | M | 42 | Negative | TR | FCD Ib+HS | 10 | B | 3 |
| 175 | M | 4 | L Temporal | RF-TC | Not available | 17 | B | 11 |
| 176 | F | 33 | Negative | RF-TC | Not available | 14 | R | 9 |
| 177 | M | 17 | Negative | RF-TC | Not available | 14 | R | 1 |
| 178 | M | 11 | L Temporal | TR | Ganglioglioma | 9 | L | 2 |
| 179 | M | 21 | Negative | TR | FCDⅠb | 15 | B | 9 |
| 180 | M | 20 | L Frontal | RF-TC | Not available | 10 | L | 3 |
| 181 | M | 54 | R Frontal | RF-TC | Not available | 15 | B | 14 |
| 182 | M | 15 | L Temporal | RF-TC | Not available | 13 | L | 1 |
| 183 | M | 31 | Negative | RF-TC | Not available | 14 | L | 1 |
| 184 | F | 20 | Negative | RF-TC | Not available | 12 | L | 11 |
| 185 | M | 20 | Negative | RF-TC | Not available | 17 | B | 5 |
| 186 | M | 6 | Negative | TR | FCDⅠb | 14 | R | 8 |
| 187 | M | 11 | Negative | TR | Unclassifiable | 13 | L | 5 |
| 188 | F | 14 | Negative | TR | FCDⅠb | 12 | L | 8 |
| 189 | M | 30 | Negative | RF-TC | Not available | 14 | L | 4 |
| 190 | M | 23 | L Hippocampus | RF-TC | Not available | 15 | B | 2 |
| 191 | M | 26 | L Hippocampus | TR | FCDⅠb | 13 | L | 3 |
| 192 | M | 14 | R Temporal-insula | RF-TC | Not available | 11 | R | 1 |
| 193 | F | 29 | B hippocampus | RF-TC | Not available | 12 | B | 2 |
| 194 | F | 41 | Negative | RF-TC | Not available | 12 | L | 4 |
| 195 | M | 16 | Negative | VNS | Not available | 9 | L | 13 |
| 196 | F | 11 | Negative | TR | FCDⅠb | 14 | L | 8 |
| 197 | F | 32 | Negative | RF-TC | Not available | 13 | L | 5 |
| 198 | F | 4 | L Temporal | RF-TC | Not available | 13 | L | 8 |
| 199 | F | 13 | Negative | RF-TC | Not available | 11 | L | 4 |
| 200 | M | 9 | Negative | TR | Unclassifiable |  |  | 11 |
| 201 | M | 16 | L Temporal-parietal | RF-TC | Not available | 11 | L | 1 |
| 202 | F | 12 | L Occipital | RF-TC | Not available | 11 | L | 2 |
| 203 | M | 14 | Negative | RF-TC | Not available | 11 | L | 2 |
| 204 | M | 21 | Negative | RF-TC | Not available | 12 | L | 10 |
| 205 | M | 10 | R Frontal | TR | FCDⅠb | 11 | R | 4 |
| 206 | M | 16 | Negative | TR+BC | Unclassifiable | 10 | L | 7 |
| 207 | M | 32 | L Frontal | / | Not available | 11 | L | 3 |
| 208 | F | 6 | Negative | / | Not available | 18 | R | 2 |
| 209 | F | 32 | Negative | RF-TC | Not available | 12 | L | 4 |
| 210 | F | 14 | L Parietal | TR | FCD Ⅰb | 8 | L | 2 |
| 211 | F | 31 | Negative | RF-TC | Not available | 13 | L | 2 |
| 212 | M | 29 | Negative | RF-TC | Not available | 11 | L | 1 |
| 213 | M | 19 | Negative | TR+BC | FCDⅠb | 12 | R | 8 |
| 214 | F | 14 | Negative | TR | FCD Ⅱb | 12 | L | 8 |
| 215 | M | 7 | Negative | RF-TC | Not available | 14 | L | 7 |
| 216 | F | 37 | Negative | RF-TC | Not available | 11 | B | 1 |
| 217 | M | 25 | R Temporal | TR+BC | FCDⅠb | 11 | R | 5 |
| 218 | F | 12 | Negative | TR | FCDⅠb | 13 | L | 5 |
| 219 | F | 16 | L Frontal | RF-TC | Not available | 7 | L | 6 |
| 220 | M | 35 | R Frontal | TR | FCDⅠb | 10 | R | 9 |
| 221 | M | 16 | L Frontal | RF-TC | Not available | 9 | L | 6 |
| 222 | M | 26 | L hippocampus | RF-TC | Not available | 12 | L | 13 |
| 223 | M | 25 | L Temporal | RF-TC | Not available | 10 | L | 14 |
| 224 | F | 7 | L Frontal | RF-TC | Not available | 10 | L | 3 |
| 225 | M | 21 | Negative | TR | FCDⅠb | 12 | R | 5 |
| 226 | M | 27 | L Hippocampus | RF-TC | Not available | 17 | B | 8 |
| 227 | M | 6 | Negative | RF-TC | Not available | 13 | R | 6 |
| 228 | F | 16 | Negative | / | Not available | 13 | R | 4 |
| 229 | M | 28 | L Frontal | RF-TC | Not available | 7 | L | 2 |
| 230 | M | 15 | Negative | TR | FCDⅠb | 11 | B | 3 |
| 231 | M | 24 | L Temporal | BC | Not available | 13 | B | 3 |
| 232 | M | 42 | Negative | RF-TC | Not available | 15 | L | 5 |
| 233 | M | 25 | Negative | TR | FCDⅡb | 8 | R | 5 |
| 234 | F | 7 | R Frontal | TD | FCD | 10 | R | 12 |

M=Male; F=Female; L=Left; R=Right; B= Bilateral; RF-TC=radiofrequency thermocoagulation;

VNS=Vagus Nerve Stimulation; BC= Bipolar electro-coagulation; TR= Tailored resection; TD=Tailored disconnection; FCD=Focal Cortical Dysplasia; HS = Hippocampal sclerosis;

GMH=gray matter heterotopia
